# Surface Modification of Sulfur-Assisted Reduced Graphene Oxide with Poly(phenylene sulfide) for Multifunctional Nanocomposites

**DOI:** 10.3390/polym14040732

**Published:** 2022-02-14

**Authors:** Minsik Choi, Junghwan Kim, Yuna Oh, Jaesang Yu, Sung-Gi Kim, Heejoun Yoo, Seongwoo Ryu, Nam-Ho You, Bon-Cheol Ku

**Affiliations:** 1 Institute of Advanced Composite Materials, Korea Institute of Science and Technology (KIST), Wanju-Gun 55324, Korea; cmin0103@kist.re.kr (M.C.); kjh4485@kist.re.kr (J.K.); t17698@kist.re.kr (Y.O.); jamesyu@kist.re.kr (J.Y.); polymer@kist.re.kr (N.-H.Y.); 2 Department of Advanced Materials Engineering, The University of Suwon, Suwon 18323, Korea; ryu@suwon.ac.kr; 3 SK Chemicals R&D Institute, Seongnam 13494, Korea; sungkikim@sk.com; 4 Grapheneall Co., Ltd., Hwaseong 12915, Korea; hjyoo1128@gmail.com; 5 Department of Nano Convergence, Jeonbuk National University, Jeonju 54896, Korea

**Keywords:** poly(phenylene sulfide) (PPS), in-situ polymerization, graphene nanocomposite, surface modification

## Abstract

The sulfur on the sulfur-assisted reduced graphene oxide (SrGO) surface provides the origin of poly(phenylene sulfide) PPS-grafting via S_N_Ar mechanism. In-situ polymerization from sulfur on SrGO afforded surface modification of SrGO, resulting in enhanced dispersibility in PPS. The tensile strength, electrical and thermal conductivities, and flame retardancy of PPS-coated SrGO were efficiently enhanced using highly concentrated SrGO and masterbatch (MB) for industrial purposes. Three-dimensional X-ray microtomography scanning revealed that diluting MB in the PPS resin afforded finely distributed SrGO across the PPS resin, compared to the aggregated state of graphene oxide. For the samples after dilution, the thermal conductivity and flame retardancy of PPS/SrGO are preserved and typically enhanced by up to 20%. The proposed PPS/SrGO MB shows potential application as an additive for reinforced PPS due to the ease of addition during the extrusion process.

## 1. Introduction

PPS is an engineering plastic that displays high chemical and thermal resistances and flame retardancy [1,2,3,4,5]. By virtue of these properties, PPS has wide applications in various cutting-edge fields, such as aircraft, aerospace, and electronics [6,7,8,9]. Many studies have been conducted to improve the electrical and thermal conductivities of PPS for a wide range of applications. To improve these properties, the development of PPS composites using carbon nanotubes or metals with high thermal or electrical conductivities has been studied [10,11,12,13,14,15,16,17]. These approaches are typically conducted through simple melt blending with components [18,19,20]. However, because melt blending has limited variables, such as the mixing ratio, temperature, and pressure, molecular design using supramolecular chemistry or covalent bonding between components has recently emerged.

As previously reported [21,22,23], the elemental sulfur-assisted reduced form (SrGO) of graphene oxide (GO), synthesized using various reducing agents, such as hydrazine, demonstrated an excellent dispersibility, enhanced electrical conductivity, and flame retardancy when compared to reduced graphene oxide (rGO) [24,25]. These properties enable SrGO to be well-dispersed in PPS and improve its thermal and electrical conductivities. In addition, the dispersibility of SrGO is further enhanced by the surface modification. Surface modification is typically performed in two ways: via non-covalent interactions, such as physical adsorption [26,27,28], and covalent bonding, such as polymer-grafting [29,30,31,32]. The latter can be more target-selective and elaborately designed based on the synthetic chemistry than the former.

The functional groups on SrGO (thiol, thioether, sulfoxide, sulfonic acid, and sulfone) can be the starting point of polymerization, considering the nucleophilic aromatic substitution (SNAr) mechanism of PPS polymerization [33,34,35,36,37]. PPS grafting from these functional groups would provide (i) enhanced dispersibility through the interaction between PPS from the matrix and composites, resulting in enhanced thermal and electrical conductivities of SrGO and the matrix [38,39,40,41], and (ii) the effective load transfer between the matrix and SrGO nanosheets, resulting in an enhanced mechanical strength [14,42,43]. Therefore, as a potential application, masterbatch (MB) of SrGO could afford a homogeneous polymer because it is well-dispersed in the polymer matrix during the melt mixing process.

Herein, PPS/SrGO composites were fabricated using in situ polymerization to form covalent bonds between PPS and SrGO, which was supported by thermogravimetric analysis (TGA) and differential scanning calorimetry (DSC). The enhanced mechanical strength and thermal and electrical conductivities of the resulting composites was demonstrated using highly concentrated SrGO MB for achieving mass production and dispersion efficiencies. SrGO was well-dispersed in the matrix when compared to the thermally reduced graphene oxide (T-rGO). This allows for the preserved or enhanced physical properties of the diluted sample compared to the as-synthesized sample. This showed the potential application of SrGO as MB for the mass production of PPS with improved physical properties.

## 2. Materials and Methods

### 2.1. Materials

Na_2_S∙5H_2_O and methyl alcohol were purchased from Daejung Chemicals (Siheung, Korea). 1,4-Dichlorobenzene was purchased from Kanto Chemicals (Tokyo, Japan). Sulfur (S_8_, powder, 98.0%) and toluene (99.5%) were purchased from Samchun Chemicals (Seoul, Korea). Lithium chloride, sodium acetate, and *N*-methyl-2-pyrrolidone (anhydrous, 99.5%) were purchased from Sigma-Aldrich. GO was obtained from Grapheneall Co., Ltd. (Hwaseong, Korea). The commercial PPS was provided by SK Chemical Co., Ltd. (Seoul, Korea).

### 2.2. Synthesis of SrGO

The synthesis of SrGO from GO followed a previously reported method [18], and the procedure is described briefly below. GO (30.0 g) and elemental S_8_ (150.0 g) were mixed thoroughly using a mechanical mixer. The mixture was then loaded into a 1 L glass reactor. The mixture was heated slightly above the melting point of elemental sulfur. After the stabilization of the mixture under these conditions, the reaction mixture was heated to 170 °C for 4 h. After cooling to 25 °C, the mixture was ground into a powder. As elemental sulfur is highly soluble in hot toluene, the powder was then added to excess boiling toluene for hot-filtration to remove residual sulfur. The mixture was filtered through a filter paper. The filtrate was yellow, and a large number of yellow crystals precipitated upon cooling. The hot-filtration process was repeated until the filtrate became colorless. In a vacuum oven at 170 °C, residual elemental sulfur was removed, affording SrGO (approximately 13 g) as a black fine powder.

### 2.3. In Situ Polymerization of PPS/SrGO and PPS/GO Composites

Prior to the polymerization of the PPS/SrGO composites, Na_2_S∙5H_2_O, NaOAc, LiCl, and *N*-methylpyrrolidone (NMP) were heated in a Parr reactor at 120 °C for dehydration under a nitrogen atmosphere. After complete dehydration, 1,4-dichlorobenzene, SrGO, and NMP were added to the mixture. The reactor was purged with nitrogen. The mixture was then stirred for 4 h. The reaction was terminated by lowering the temperature of the reactor to room temperature. The product was filtered through a membrane filter and washed repeatedly with hot deionized water and methyl alcohol. The residue was dried overnight in a vacuum oven at 100 °C. To confirm the filler effect of SrGO, a similar reaction was performed using GO instead of SrGO as a control, referred to as PPS/GO.

### 2.4. Preparation of Diluted PPS Composites by MB

A twin-screw extruder was used to dilute the highly concentrated MB with PPS. First, according to the ratio, 150 g of PPS in pellet form and MB in the powder form were placed in a bag and prepared. The MB powder was hand mixed to ensure that it was evenly coated on the prepared PPS pellets. The prepared mixture was gradually added to the twin-screw extruder through the hopper. Extrusion was carried out at a screw speed of 70 rpm and a heating zone temperature of 270 °C. The extruded material was immersed in an ice bath for coagulation after being extruded through the die. Thereafter, pellet-type samples were obtained by pelletizing.

### 2.5. Classical Micromechanics for Effective Thermal Conductivity (TC) 

The Mori-Tanaka method (MTM) considers a single ellipsoidal heterogeneity embedded within an infinite homogeneous matrix domain subjected to a constant far field heat flux, as applied to steady-state heat conduction problems. The MTM takes the opposite view and uses the continuum averaged heat flux vector (*q*) and temperature gradient (∇T) to predict the effective TC tensor for the composite. The heat flow in a composite may be characterized in terms of the far field applied heat flux vector (*q*) as follows:

(1) $$q=-\bar{K}\cdot \nabla T$$

where $\bar{K}$ represents the effective second-rank TC tensor and ∇T represents the continuum averaged temperature gradient. It is assumed that the matrix contains m distinct types of ellipsoidal heterogeneities (*p* = 1, 2, …, *m*), each consisting of np layers (*α_p_* = 1, 2, …, *n_p_*; *p* = 1, 2, …, *m*). Each type of heterogeneity has distinct thermal properties, shapes, and orientation distributions. In this case, the effective TC tensor $\bar{K}$ can be expressed as follows:

(2) $$\bar{K}=K_{\left(0\right)}\cdot \{I+\overset{m}{\underset{p=1}{\sum }}[\overset{n_{p}}{\underset{\alpha_{p}=1}{\sum }}c_{{\left(p\right)}_{\alpha_{p}}}\left(S_{\left(p\right)}-I\right)\cdot \left(A_{\left(p\right)}{}^{\left(\alpha_{p}\right)}-S_{\left(p\right)}{)}^{-1}\right]\}\cdot \;\{I+\overset{m}{\underset{p=1}{\sum }}{\left[\overset{n_{p}}{\underset{\alpha_{p}=1}{\sum }}c_{{\left(p\right)}_{\alpha_{p}}}S_{\left(p\right)}\cdot \left(A_{\left(p\right)}{}^{\left(\alpha_{p}\right)}-S_{\left(p\right)}{)}^{-1}\right]\right\}}^{-1}$$

where

(3) $$A_{\left(p\right)}{}^{\left(\alpha_{p}\right)}={\left(K_{\left(0\right)}-K_{\left(p\right)}{}^{\left(\alpha_{p}\right)}\right)}^{-1}\cdot K_{\left(0\right)}$$

*I* represents the second-rank temperature gradient concentration tensor for the *α_p_*^th^ layer of the *p*^th^ heterogeneity (*α_p_* = 1, 2, …, *n_p_*, *p* = 1, 2, …, *m*); $K_{\left(p\right)}{}^{\left(\alpha_{p}\right)}$ is the second-rank TC tensor for the *α_p_*^th^ layer of the *p*^th^ heterogeneity; *S*_(_*_p_*_)_ represents the volume fraction of the *α_p_*^th^ layer of the *p*^th^ heterogeneity, and *S*_(*p*)_ represents the second-rank Eshelby tensor common to the *p*^th^ heterogeneity and all of its layers.

### 2.6. Characterization

To evaluate the reduction degree of SrGO, TGA was conducted using a TGA Q50 instrument (TA Instruments, New Castle, DE, USA) at a temperature range of 50–900 °C and a heating rate of 10 °C/min under an N_2_ atmosphere. X-ray photoelectron spectroscopy (XPS) spectra were obtained using a Thermo Scientific K-alpha (Thermo VG, Waltham, MA, USA) with monochromated Al Kα (1486.6 eV). The X-ray diffraction (XRD) patterns of GO and SrGO were obtained using an X-ray diffractometer (D/MAX 2500, Rigaku) with Cu Kα radiation (k = 0.15418 nm) at 40 kV and 50 mA. The electrical conductivity was measured using an HPRM-A2 powder resistivity measurement system at a load of 2000 kg. Raman spectra were collected on a multidimensional liquid-NIR laser Raman analyzer (inVia reflex) with excitation by an incident laser at 514.5 nm. The thermal properties of the PPS composites were evaluated using DSC performed with a DSC Q20 instrument analyzer (TA Instruments, New Castle, DE, USA) at a temperature range of 50–400 °C under an N_2_ flow at a heating rate of 10 °C/min. High-resolution transmission electron microscopy (HR-TEM) analysis was conducted on a Titan G2 Cube 60–300 system operating at 80 kV. For the tensile test of the diluted PPS composite, a dog-bone specimen was manufactured based on the ASTM D638 (Type V) standard using an injection molding machine. For mechanical characterization, all samples were characterized using a universal testing machine (UTM) with a 3 kN load cell at a loading rate of 1 mm/min. The isotropic TC was measured using the transient plane source method (Hot-disk AB, TPS-2500s). The heat release capacity (HRC) was obtained using a pyrolysis combustion flow calorimeter (Fire Testing Technology Ltd., East Grinstead, UK) on samples of approximately 10 mg, which followed the principle of oxygen consumption calorimetry. The samples were heated to 900 °C at a heating rate of 1 °C/s under an atmosphere of N_2_:O_2_ = 80:20 at a flow rate of 100 cc/min. The electrical conductivities of the PPS composites were measured using an automatic sheet resistance measurement system (ARMS-600). The three-dimensional X-ray microtomography (3D XRM) images were obtained using a ZEISS Xradia Ultra/Versa hybrid system at a power of 5 W and 50 keV.

## 3. Results

### 3.1. Preparation of SrGO

**Scheme 1:**
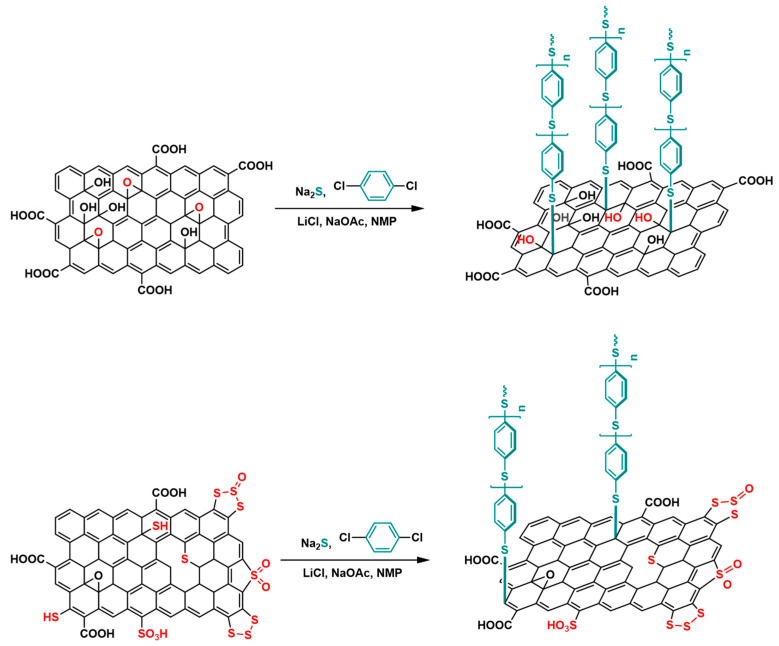
Preparation of PPS/GO and PPS/SrGO composites.

SrGO is reduced graphene containing sulfur functional groups, such as thiol, thioether, and sulfoxide. Compared to other chemically reduced GO, SrGO has higher electrical and thermal conductivities and higher dispersibility in organic solvents [21,22]. In this study, SrGO was grafted with PPS using in situ polymerization under typical PPS polymerization conditions (Scheme 1). PPS/GO composites were also prepared to compare the physical properties of the composites.

**Figure 1:**
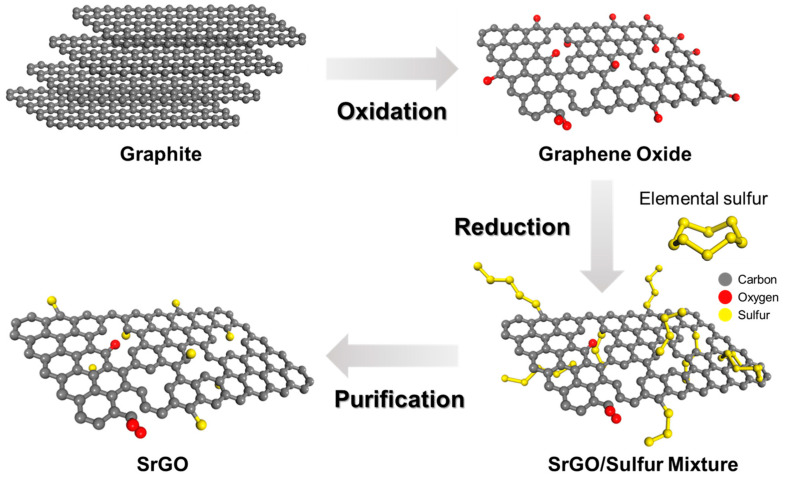
Synthetic route to prepare SrGO from graphite using elemental sulfur.

In this study, SrGO was prepared according to a previously reported procedure (Figure 1) using elemental sulfur as a reducing agent, except the purification method [21]. The removal of residual sulfur by sonication in toluene is inefficient for large-scale synthesis (>30 g of GO). This is because (i) excessive toluene is used to dissolve sulfur, and (ii) long-lasting sonication degrades SrGO. To avoid these issues, a hot-filtration method in toluene was adopted with minimal sonication. As a result, SrGO without residual elemental sulfur was obtained in a moderate yield. 

**Figure 2:**
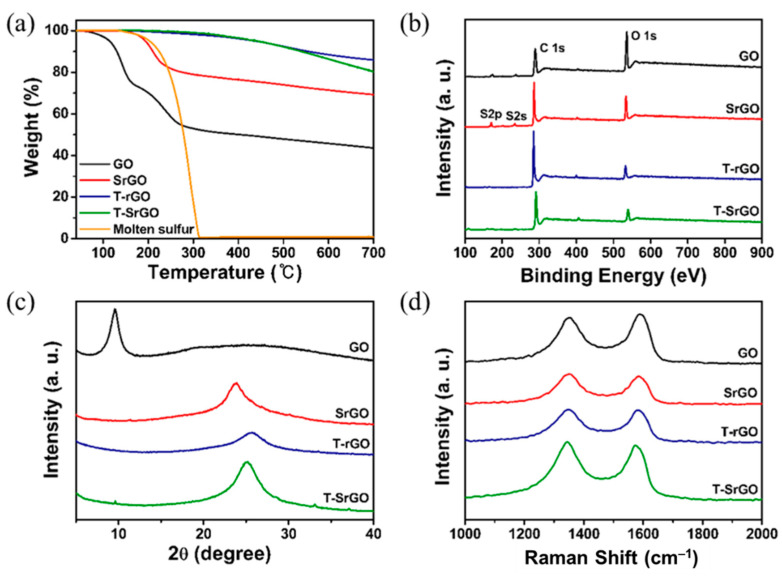
Characterizations of GO, SrGO, T-rGO, and T-SrGO. (**a**) Thermogravimetric analysis (TGA) curves of GO, SrGO, T-rGO, T-SrGO, and molten sulfur. (**b**) X-ray photoelectron spectroscopy (XPS) spectra of GO, SrGO, T-rGO, and T-SrGO. C1s, O1s, S2s, and S2p regions are shown. (**c**) X-ray diffraction (XRD) patterns and (**d**) Raman spectra of GO, SrGO, T-rGO, and T-SrGO.

The absence of elemental sulfur and the development of sulfur-containing functional groups were confirmed using the TGA (Figure 2a) and XPS (Figure 2b) of molten sulfur and SrGO. Under high temperature polymerization conditions, GO is assumed to be thermally reduced to form T-rGO [44,45]. To investigate the reduction of GO, GO was heated in NMP under the same polymerization temperature and pressure. The decomposition temperature at 5% weight loss (T_d5_), char yield, and C/O ratio of T-rGO indicated the reduction of GO, as shown in Figure 2 and Table 1. This was also cross-checked using powder XRD (Figure 2c). The XRD spectrum of GO shows an intense characteristic peak at 2*θ* = 9.5°, which corresponds to an interlayer distance of approximately 9.3 Å. Meanwhile, the peak of T-rGO was shifted to a wide angle, indicating a narrow interlayer distance (approximately 3.46 Å). This suggests that the GO was fully reduced by thermal treatment. The higher interlayer distance of SrGO (3.72 Å) than that of GO was due to sulfur-containing functional groups on the SrGO sheet. In the Raman spectra, the intensity ratio of G and D bands, I_G_/I_D_ of GO, T-rGO, and T-SrGO (thermally-treated SrGO under in-situ polymerization condition) were 0.86, 1.04, and 1.06, respectively (Figure 2d and Table 1). These data indicate that GO was solvothermally reduced during polymerization.

### 3.2. In-Situ Polymerization and Characterization of PPS/GO and PPS/SrGO Composites

After the identification of SrGO, PPS/GO and PPS/SrGO composites were prepared using an in situ polymerization method. The method is described in Scheme 1, in which PPS grows from nucleophilic functional groups on GO or SrGO sheets. The in situ polymerization was executed under the same conditions typical of PPS polymerization. For typical PPS polymerization, S^2-^ is polymerized with p-dichlorobenzene through S_N_Ar. Similarly, nucleophilic elements on SrGO, such as S^-^ from thiol, participate in polymerization through the S_N_Ar mechanism. In addition, sulfide as a PPS end group facilitate epoxide opening with nucleophilic attack [27]. As a result, covalent bonds exist between the PPS polymer and GO or SrGO.

**Figure 3:**
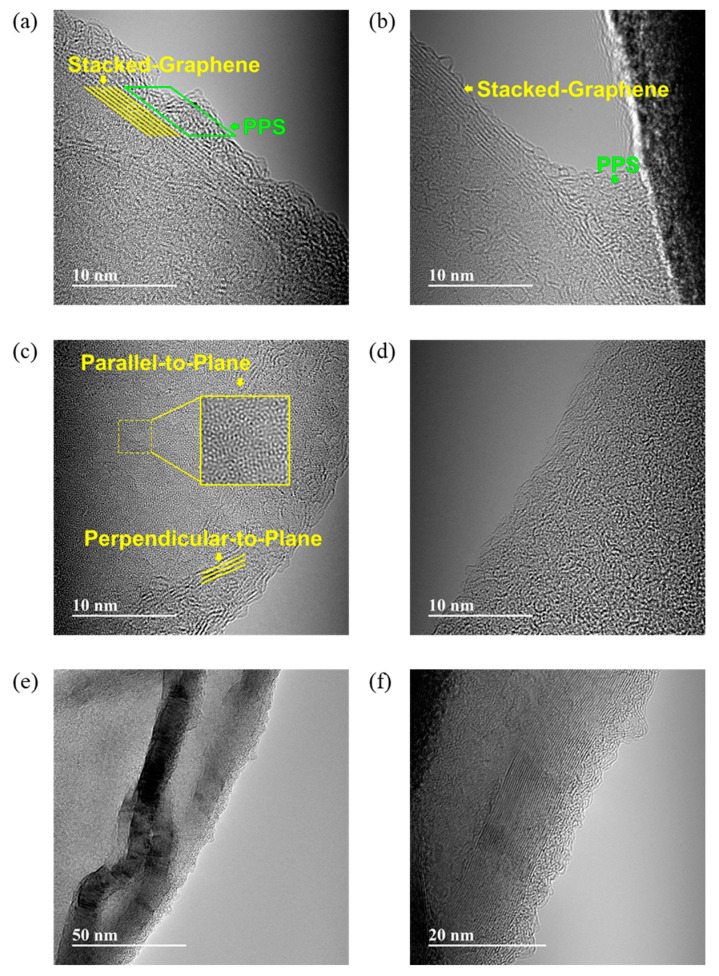
TEM images of PPS/SrGO and PPS/GO composites. (**a**,**b**) stacked-graphene with surface modification by PPS from the side, (**c**) Domains of stacked-graphene being parallel- or perpendicular-to-plane, (**d**) Pristine PPS, (**e**,**f**) PPS/GO composite.

Morphological evidence of incorporation between PPS and SrGO was provided by TEM monitoring of the PPS/SrGO composite (2:1 wt%) (Figure 3). Specimens for the TEM analysis were prepared by dispersing the PPS/SrGO composites, R_S,50_, in EtOH with sonication for 1 h, then dropping the dispersion onto a grid and drying the sample in vacuum. As shown in Figure 3a,b, the PPS domain was fused to the stacked graphene layer (the thick and dark layer of Figure 3b on the right-hand side is a bulk PPS/SrGO composite, which is too thick to identify the structure using TEM). The graphene-PPS fused-structure constitutes most of the material. The naked graphitic domain is shown in Figure 3c and comprises a hexagonal-repeating structure (parallel to the plane) and a stacked layer structure (perpendicular to the plane). Solely entangled polymer regions are shown in Figure 3d. These TEM images indicate that PPS is grown on the surface by coating it. In addition, PPS on the surface can connect SrGO to the pristine PPS resin (dark region on the right side), as shown in Figure 3b. This increases the miscibility and dispersibility of PPS/SrGO composite in the pristine PPS resin during the in situ polymerization or melt blending process. For the comparison, morphology of PPS/GO composites was monitored by TEM as shown in Figure 3e,f. Figure 3e shows the high contrast between PPS and graphite domains. The magnified image, Figure 3f, clearly demonstrates the thicker and denser graphitic layers of PPS/GO than those of PPS/SrGO. Comparison of Figure 3a–d and Figure 3e,f indicates that SrGO is well-dispersed in PPS resin and finely integrated with the PPS with fewer layers.

**Figure 4:**
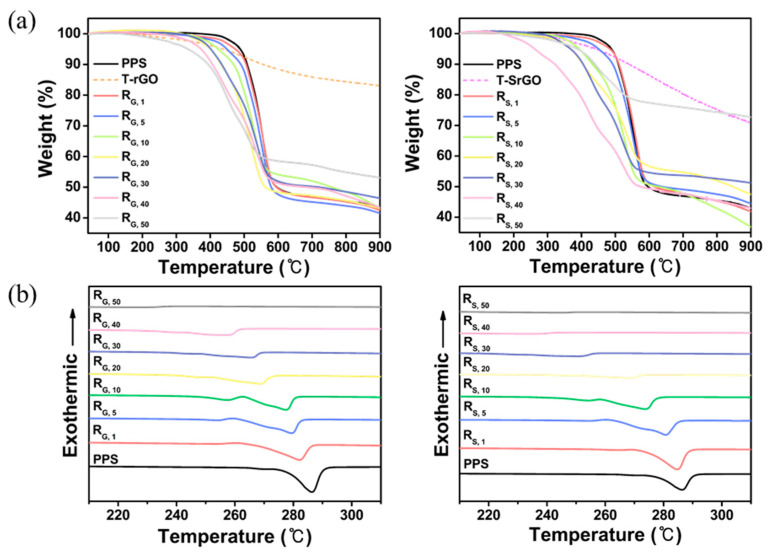
(**a**) TGA and (**b**) differential scanning calorimetry (DSC) curves of PPS/GO (**Left**) and PPS/SrGO (**Right**) composites. (R_x,y_—composite sample of ratio 100:y between PPS and x. For GO, x = G and for SrGO, x = S).

TGA and DSC curves and their details for PPS/GO and PPS/SrGO are shown in Figure 4a,b and Table 2, respectively. In Figure 4a, there are two characteristic decompositions: the initial decomposition of PPS started at 450 °C, and the thermal decomposition of T-rGO (or T-SrGO) was observed at 300–900 °C. Following the simple rule of mixtures, the curves gradually change from the PPS to GO as the weight percentage of GO increases. Meanwhile, for the case of SrGO, the curve of R_S,50_ deviates from the expected pattern of the rule of mixtures.

In this sample, it was hypothesized that the thiol group from excess SrGO in a polymerization reactor decrease the monomer ratio of [DCB]/[Na_2_S] in the solution. This leads to a low molecular weight of PPS, which is soluble in a solvent, resulting in the loss of PPS during the filtration process. The insoluble T-SrGO was preserved in the reaction and filtration processes. Instead, the preserved PPS is mostly bound to the surface of SrGO, as shown in the TEM image of R_S,50_. Although short PPS can weaken the mechanical properties of the diluted PPS resin, it could enhance the dispersibility of PPS/SrGO composites as surface modification.

In industry, compounding with a small amount of additive MB could improve the properties of thermoplastics. Typically, MB has relatively high concentrations of additives to minimize the feeding amount of composites and reduce manufacturing costs. From the highest char yield shown in the TGA curves, R_S,50_ was expected to have the highest concentration of graphenic materials. In addition, R_S,50_ exhibited an enhanced solubility through successful surface modification with PPS. Thus, the desired level of physical properties could be achieved with a minimal amount of R_S,50_ as MB, without the significant degradation of the mechanical properties.

In Figure 4b, the peak of the DSC curve of pristine PPS indicates a melting point of 286.4 °C for PPS. With increase in GO or SrGO contents in the composites, the peaks corresponding to the melting point are shifted to low temperatures, probably because of the low packing density and molecular weight of the polymer chains because of GO and SrGO.

### 3.3. Physical Properties of PPS/GO and PPS/SrGO Composites

**Figure 5:**
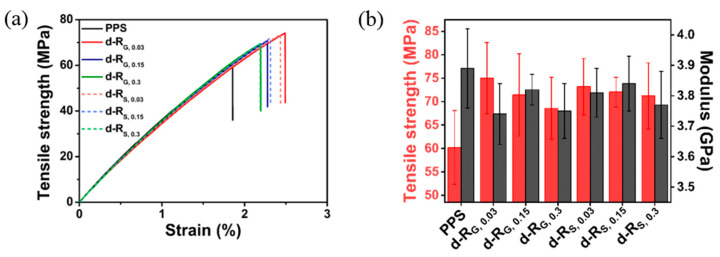
(**a**) Representative stress-strain curves of each PPS/SrGO composite, (**b**) mechanical properties of PPS/SrGO composites with different filler contents. (d-R_x,y_—diluted composite samples of ratio 100:y between PPS and x. For GO, x = G and for SrGO, x = S).

The mechanical properties of the PPS/GO and PPS/SrGO composites were measured using a sample prepared by the dilution of R_G,50_ and R_S,50_ as the MB. The samples with GO or SrGO concentrations of 0.03, 0.15, and 0.3 wt% (labeled d-R_x,y_) were prepared by extruding and injection molding. The tensile strength of the composites displayed a remarkable increase compared to the pristine PPS sample (up to 25%) (Figure 5a,b, Table 3). The degree of increase in the tensile strength is the highest at 0.03 wt% of GO or SrGO and decreases with a higher GO or SrGO content. This implies that a small quantity of GO or SrGO (<0.03 wt%) could improve the mechanical strength. The enhancement in the mechanical strength could be attributed to the formation of covalent bonds between graphene (GO and SrGO) and polymers (PPS), which afford an effective load transfer with increased elongation. However, as the filler content increased above 0.03 wt%, the filler could interrupt the crystallization of the polymer strands and form self-aggregation, thereby reducing the mechanical strength. Meanwhile, the moduli of the samples showed slight fluctuations. The reason is presumed to be that the elastic modulus is mainly derived from PPS, which accounts for most of the material. The elastic modulus evaluates the elasticity of rigid or solid materials, as its value is calculated by dividing the deformation of a material by the power required to deform it. For PPS/SrGO composites, elasticity is mainly originated from PPS, as its single strand can be considered as a coiled spring. Compared to PPS, SrGO or GO have a two-dimensional structure, which has less structural change and entanglement. Furthermore, the chain length of PPS synthesized by in-situ polymerization is shorter than that of pristine PPS. This can have a detrimental effect on the tensile modulus. In addition, the graphenic contents are too small (maximum 0.3 wt%) to make a remarkable increase in the elastic modulus, as we reported in the literature [29]. Therefore, regardless of graphenic materials, the moduli of the composites are similar to polymer itself and its composites.

**Figure 6:**
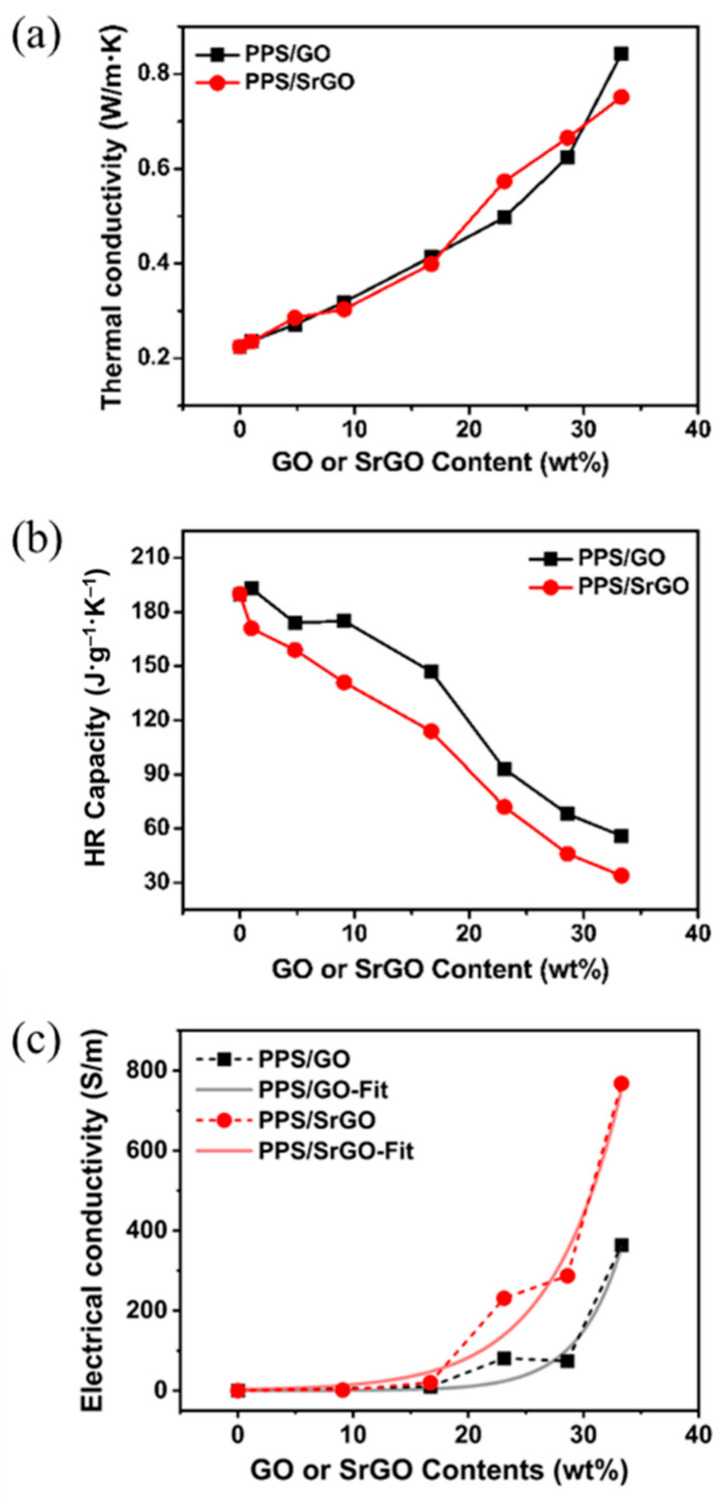
(**a**) Thermal conductivity (TC), (**b**) heat release capacity (HRC), and (**c**) electrical conductivity of PPS/GO and PPS/SrGO composites.

To evaluate the effects of GO or SrGO on PPS composites, several experiments were conducted to measure the physical properties as shown in Figure 6. First, the TC of the composites were measured (Figure 6a). Despite the fluctuation, the TC of the GO and SrGO composites were similar and appeared to increase linearly or in a sigmoidal fashion. For R_G,50_ and R_S,50_ samples, the TC increased by 375% and 335%, respectively. Second, the HRC was measured to evaluate the flame retardancy (Figure 6b). As SrGO was reported to have a high flame retardancy [22], the PPS/SrGO composite showed a lower HRC (34 J·g^−1^·K^−1^) than the PPS/GO composite, which was 82% lower than that of the pristine PPS resin. The electrical conductivity of the composites were also measured (Figure 6c). As it was impossible to measure the electrical conductivity using our resistivity measurement system for the composites containing a small portion of GO (R_G,1_–R_G,10_) or SrGO (R_S,1_–R_S,5_), a guide line was added to identify the sigmoidal pattern of the electrical conductivity. The values obtained for R_G,50_ (364 S/m) and R_S,50_ (768 S/m) are similar to those of pristine T-rGO (430 S/m) and T-SrGO (866 S/m), which indicate the premature saturation of the electrical conductivity. This might be due to the synergistic effects of PPS and GO or SrGO on electrical conductivity.

**Figure 7:**
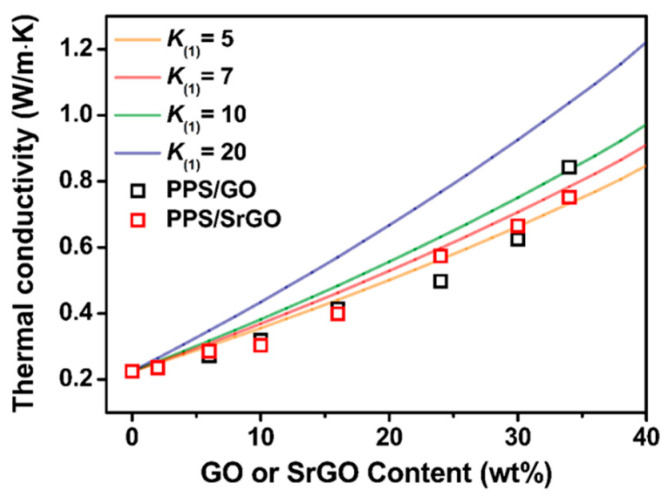
Predicted TC of PPS/GO and PPS/SrGO using MTM simulation method.

In this study, the MTM developed previously [46,47,48] was used to predict the effective TCs of PPS composites containing GO and SrGO. The GO and SrGO aspect ratio (the ratio of diameter to thickness), L/D = 1000, was used. The width and thickness of the GO used were 5.0 and 0.35 um, respectively. Figure 7 shows the experimentally measured TCs of composites comprising PPS (*K*_(0)_ = 0.2246 W/m∙K) reinforced with 3D, randomly oriented fillers (*K*_(1)_ = 5, 7, 10, and 20 W/m∙K) as a function of the filler weight fraction. The TCs of the bulk composites increased with the increasing filler content. With small amounts of conductive filler materials, the thermal properties of the PPS polymer composites were slightly improved. However, the effective TCs of PPS composites with large filler concentrations increased significantly when compared to those of PPS composites with relatively low weight fractions of fillers. The estimated thermal properties of PPS composites containing fillers with relatively high thermal values (*K*_(1)_ = 10 and 20 W/m∙K) are substantially better compared to the experimental data. In contrast, the predicted TCs of PPS composites containing fillers with relatively low thermal values (*K*_(1)_ = 5 and 7 W/m∙K) were in good agreement with the experimentally measured data, as shown in Figure 6. The MTM model was considered as a relatively accurate predictor of the experimental results over a range of filler weight fractions with proper thermal values of fillers.

### 3.4. Application of PPS/SrGO Composite as MB Using High Dispersibility of SrGO

The masterbatch is used for convenience in the process in industry, because the desired additive is pre-dispersed in the polymer matrix. Simple melt compounding with MB provides the resulting polymer containing the finely-dispersed additive. Therefore, it is important to fabricate highly concentrated and dispersed MBs. However, graphenic materials have very low dispersibility and generally need chemical modification such as oxidation. Unfortunately, chemical modifications can deteriorate the physical properties of graphenic materials and graphene-dispersed polymers. In addition, the dispersion matrix could negatively affect the physical properties of MB-diluted polymer. Considering this possibility, it is difficult for graphene-dispersed polymers to maintain or improve physical properties compared to the as-synthesized polymers. Nevertheless, the PPS/SrGO composites in this study were expected to show maintained or enhanced performance for the MB-diluted PPS compared to the as-synthesized PPS, because the successful surface coating of SrGO with PPS could enhance the dispersibility of SrGO. 

**Figure 8:**
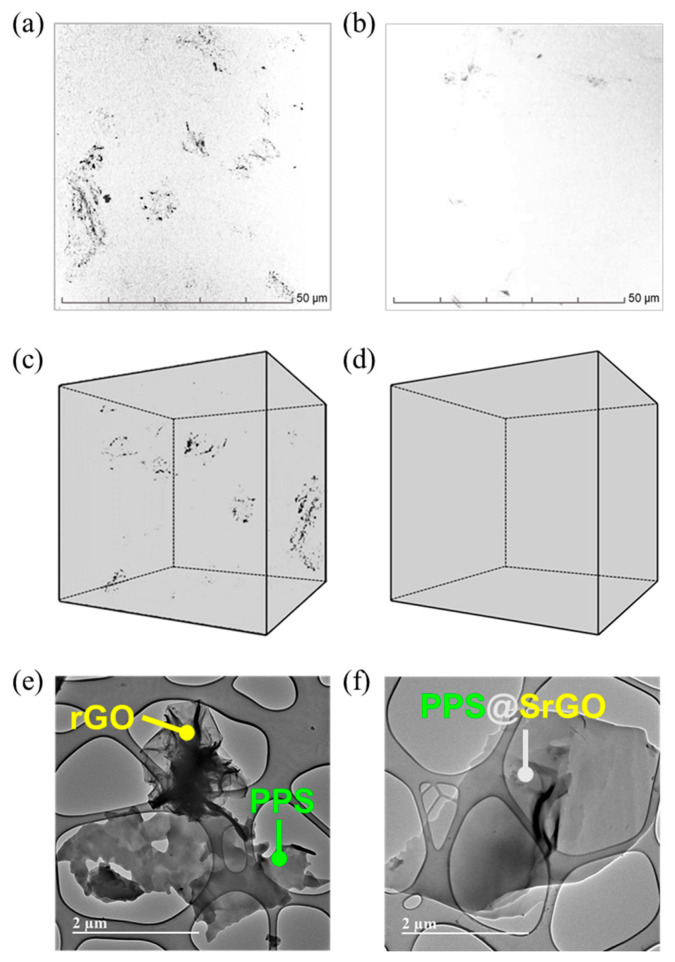
X-ray computed tomography (CT) images for 2D-scanning of (**a**) PPS/GO and (**b**) PPS/SrGO, and 3D-scanning image of (**c**) PPS/GO and (**d**) PPS/SrGO within a cubic space. d-R_G,0.5_ and d-R_S,0.5_ were used, TEM images for bulk PPS composites of (**e**) PPS/GO and (**f**) PPS/SrGO.

SrGO was expected to exhibit a high dispersibility in the PPS matrix [22]. In addition, PPS-coated SrGO would provide further enhancement of dispersibility. These would provide a significant increase in the physical properties of the PPS/SrGO composites compared to the pristine PPS, even at low concentrations of SrGO. Therefore, an XRM experiment was conducted to confirm whether SrGO was well-distributed across the composite, as shown in Figure 8. For two-dimensional (2D)-scanning images shown in Figure 8a,b, PPS/GO depicted as shaded spots is agglomerated into several groups, indicating a low dispersibility in the PPS resin. On the contrary, the image of PPS/SrGO shows few spots, confirming the high dispersibility of the PPS resin. In addition, the 3D-scanning images (Figure 8c,d) demonstrate the same results as the 2D images for dispersibility.

As shown in Figure 8e, domains of PPS and rGO (thermally reduced GO during in-situ polymerization) in the PPS/GO composites are fully divided, which are indicated by their respective arrows. The domains of rGO are corrugated and folded like thin paper, whereas the domains of PPS are irregular and mottled. In contrast, the PPS/SrGO composite in Figure 8f shows a homogeneous appearance. This indicates the successful surface coating with PPS throughout the bulk material, which can be cross-checked by the magnified TEM images in Figure 3. The rGO in the PPS/GO composites is densely packed and shown as the dark and opaque region. Those regions appear as dark spots in the X-ray CT images in Figure 8c.

**Figure 9:**
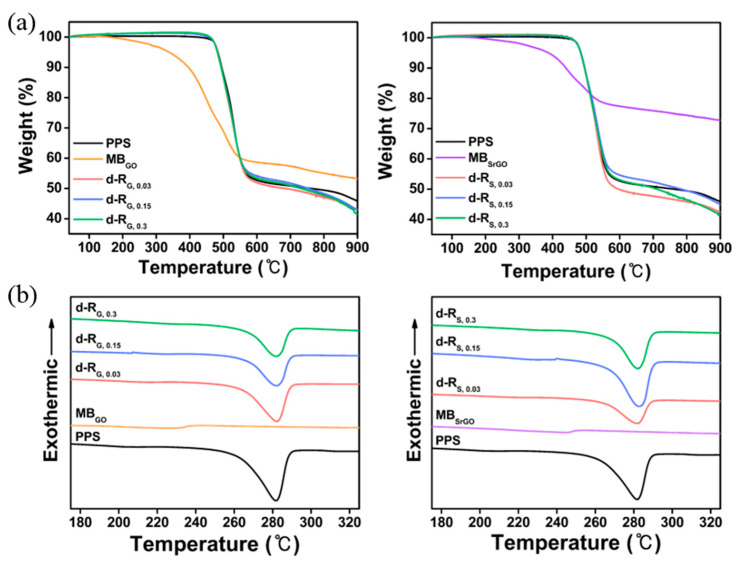
(**a**) TGA curves and (**b**) DSC curves of PPS-diluted composites. (**Left**—PPS/GO and **right**—PPS/SrGO).

Based on the high dispersibility of SrGO, diluted samples using GO MB (d-R_G,0.1_, d-R_G,0.5_, and d-R_G,1.0_) and SrGO MB (d-R_S,0.1_, d-R_S,0.5_, and d-R_S,1.0_) were prepared. In Figure 9a, the TGA curves of the samples demonstrate slight deviations between the curves of the pristine PPS and diluted samples because of the relatively small portion of GO or SrGO compared to PPS. For the same reason, the Tm values of the diluted samples and pristine PPS are similar at approximately 282 °C (Figure 9b and Table 4).

**Figure 10:**
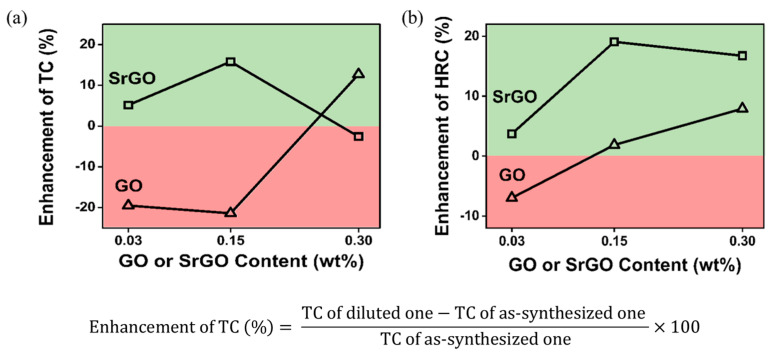
Enhancements of (**a**) TC and (**b**) HRC of PPS-diluted composites compared to pristine PPS (0.03, 0.15, and 0.3 wt% of GO and SrGO). Enhancements of TC and HRC are shown in green and vice versa in red. Enhancement of TC was calculated following the equation described above. Calculation of enhancement of HRC is conducted in the same way.

As the high dispersibility of SrGO was confirmed using XRM, the TC and HRC between the as-synthesized and diluted composites using MB were compared. Their enhancement after dilution was calculated, as shown in Figure 10. In Figure 10a, at low concentrations, the TC of GO decreases at d-R_G,0.1_ and d-R_G,0.5_, whereas the TC of SrGO increases or conserves at all points. Similarly, the enhancement of HRC in Figure 10b increases or conserves SrGO by approximately 20%. However, for GO composites, HRC deteriorated at 0.03 wt% of GO (d-R_G,0.1_) and the enhancement of HRC was lower than that of SrGO at other points. 

In comparison to the case of GO, the dilution of MB_SrGO_ enhanced or at least preserved the TC and flame retardancy. This can be attributed to the higher concentration of SrGO than GO in masterbatch at polymerization temperature, 260 °C, as shown in the TGA curves (Figure 4a), and the positive correlation between the physical properties and graphenic contents (Figure 6). Therefore, when the same weight of MBs are used, MB of SrGO contains more graphenic materials, which can result in a significant improvement in physical properties. Although more graphenic materials in the MB of SrGO were unintentionally developed, the high concentration of SrGO in MB is advantageous in that the amount of short PPS synthesized during the in situ polymerization and the MB are minimized. As short PPS is detrimental to physical properties, MB of SrGO helped to improve the physical properties more effectively than that of GO.

## 4. Conclusions

In summary, grafting PPS from the sulfur on SrGO surface provided improved dispersibility of SrGO in PPS. Sulfur on the SrGO surface participated in PPS-polymerization via S_N_Ar mechanism, as either a starting point or end point. The surface-modified SrGO contributed to the increased load transfer and miscibility between PPS and SrGO. The composites showed enhanced tensile strengths, electrical and thermal conductivities, and flame retardancies when compared to a pristine PPS polymer. Diluted samples were prepared using highly concentrated composites, mainly one-third of SrGO or GO, as MB. The high dispersibility of SrGO in the PPS resin compared to GO was visually demonstrated using the XRM technique. The diluted samples of PPS/SrGO composites presented preserved or improved physical properties, even in highly diluted samples when compared to the composites of GO. These properties make composites suitable for MB to enhance the physical properties of the PPS resin. The comparison with PPS/GO composites demonstrated that the MB of SrGO enhanced or preserved the physical properties after dilution with PPS resin, implying that the MB is economical and effective for application in industrial purposes. There are limitations of (i) shortened PPS in MB, (ii) purification issue of removing unbound PPS, and (iii) unclear evidence for covalent linkage between PPS and SrGO. Efforts are currently underway to optimize in situ polymerization of MB and expand the scope to other polymers for versatility.

## Figures and Tables

**Table 1:** Details of TGA, XPS, and XRD.

Sample	T_d5_ (°C)	Char Yield (%)	Atomic Percent			C/O	I_G_/I_D_	d-Spacing (Å)	Electrical Conductivity (S/m)
			C (%)	O (%)	S (%)				
GO	114.2	43.6	67.6	32.4	-	2.1	0.86	9.25	-
SrGO	193.9	69.2	76.4	20.6	3.0	3.7	1.08	3.72	442
T-rGO	438.2	85.9	88.8	11.2	-	8.0	1.04	3.46	430
T-SrGO	441.6	80.2	94.5	3.7	1.8	25.4	1.06	3.54	866

**Table 2:** Details of TGA and DSC graphs of PPS/GO and PPS/SrGO composites.

Ratio		PPS	R_1_	R_5_	R_10_	R_20_	R_30_	R_40_	R_50_
PPS/GO	T_d5_ (°C)	488.3	474.1	449.1	423.3	400.2	401.8	372.7	338.4
	Char Yield (%)	42.9	42.5	41.5	43.2	43.1	46.4	43.4	53.2
	T_m_ (°C)	286.4	284.6	279.4	277.5	269.1	265.9	257.1	226.2
PPS/SrGO	T_d5_ (°C)	488.3	484.0	450.3	392.8	368.7	367.4	262.1	376.8
	Char Yield (%)	42.9	41.9	44.4	36.8	47.5	51.2	42.9	72.1
	T_m_ (°C)	286.4	284.6	280.9	273.7	269.2	250.6	234.4	244.5

**Table 3:** Details of mechanical properties of PPS/GO and PPS/SrGO composites.

Filler Contents (wt%)		0	0.03	0.15	0.3
PPS/GO	Tensile Strength (MPa)	60.19 ± 7.78	74.98 ± 7.51	71.41 ± 8.91	68.57 ± 6.45
	Modulus (GPa)	3.89 ± 0.13	3.74 ± 0.10	3.82 ± 0.05	3.75 ± 0.09
	Elongation (%)	1.72 ± 0.61	2.40 ± 0.71	2.28 ± 0.47	2.23 ± 0.26
PPS/SrGO	Tensile Strength (MPa)	60.19 ± 15.56	73.2 ± 11.88	72.04 ± 5.94	71.22 ± 13.58
	Modulus (GPa)	3.89 ± 0.13	3.81 ± 0.08	3.84 ± 0.09	3.77 ± 0.11
	Elongation (%)	1.72 ± 0.61	2.28 ± 0.51	2.37 ± 0.33	2.30 ± 0.49

**Table 4:** Details of thermal properties of PPS-diluted composites.

Filler Content (wt%)		0	0.03	0.15	0.3
PPS/GO	T_d5_ (°C)	483.9	483.2	482.5	482.2
	Char Yield (%)	45.9	43.0	42.9	41.5
	T_c_ (°C)	199.1	220.3	216.4	208.2
	T_m_ (°C)	282.0	282.2	282.3	282.3
PPS/SrGO	T_d5_ (°C)	483.9	483.7	484.9	483.8
	Char Yield (%)	45.9	42.3	44.9	41.4
	T_c_ (°C)	199.1	211.9	219.7	223.3
	T_m_ (°C)	282.0	282.0	283.0	282.3

## Data Availability

All data produced in this study are presented in this paper.
